# Books and Authors

**Published:** 1908-09

**Authors:** 


### BOOKS AND AUTHORS.

---

**Modern Medicine.** Its Theory and Practice. Edited by **William Osler,** M.D., Regius Professor of Medicine in Oxford University, Eng. Assisted by Thomas McCrea, M.D., Associate Professor of Medicine and Clinical Therapeutics in Johns Hopkins University, Baltimore. In seven octavo volumes of about 900 pages each, illustrated. Volume IV. Lea & Febiger: Philadelphia and New York, 1908. (Cloth, $6.00, leather, $7.00, half morocco, $7.50 net.)

The contents of this volume include diseases of the circulatory system, diseases of the blood, and diseases of the spleen, thymus, and lymphatic glands. These topics are of particular interest to clinicians and are set forth by men of capability. The first part, dealing with diseases of the circulatory system, constitutes about two-thirds of the book, and is an exhaustive presentation of the subject. Less than one hundred and fifty pages are allotted to diseases of the blood, though the more important features relating to this topic are taken up. About one hundred pages are devoted to the consideration of diseases of the spleen, thymus and lymph glands. The chapter relating to the spleen is prepared by Irving Phillips Lyon of Buffalo, and is a careful résumé of the literature of the subject. The other chapters in this section—diseases of the thymus, and diseases of the lymphatic glands—are supplied by Alfred Scott Warthin, of Ann Arbor, and these chapters also present the latest knowledge on the subjects dealt with.

This volume is replete with valuable material and stands well up to the level of the previous numbers of the series. We are at a loss, however, to understand why the editor permits the contributors habitually to end sentences with the abbreviation "etc."—or again to use the abbreviation viz. for "namely"—or still again to make use of diphthongs. These incongruities serve to mar, every now and then, what otherwise would be a most perfect page.

---

**Medical Gynecology.** By **Samuel Wyllis Bandler,** M.D., Adjunct professor of diseases of women, New York Post-Graduate Medical School and Hospital; associate attending gynecologist to the Beth Israel Hospital, New York. Octavo, pp. 676. With original illustrations. Philadelphia and London: W. B. Saunders Co. 1908. (Cloth, $5.00).

Many attempts have been made in recent years to deal with gynecology from the medical point of view, that is to say, to publish a treatise on gynecology from the non-surgical side of the subject. Some of the works have presented the topic with a reasonable success, but in general they have failed to obtain the confidence of the profession and have passed from view.

This work is the product of the author's clinical experience as a teacher and is a practical application thereof to the needs of the every-day practitioner. The author's directions for examination are thoroughly scientific in character and, besides, are so well illustrated that even the novitiate may thereby learn the technic. So, too, with the methods employed by Bandler in medical treatment, the description of which occupies the second section of the book. The employment of pessaries in appropriate conditions will ever be a topic of interest, because to oppose a mechanical device against a mechanical defect involves an interesting principle,—one quite as applicable to the human body as elsewhere. The author is particular in his directions for the employment of pessaries, felicitous in his descriptions as to their introduction, and the illustrations give clearness as to his meaning.

The causes and treatment of erosions of the cervix of inflammatory conditions of the genital tract, and of the endometrium, of pelvic peritonitis, and subinvolution all receive due attention. The several phases of uterine displacements are dealt with in an interesting and instructive manner. Some of the topics need elaboration which doubtless will be done in a subsequent edition, but taken as a whole the book presents the subject of medical gynecology to the general practitioner in a more satisfactory fashion than has been done by any previous work that has come within our view.

---

**The Development of Ophthalmology in America, 1800-1870.** A Contribution to Ophthalmic History and Biography. An address delivered in abstract before the section of ophthalmology of the American Medical Association, June 4, 1907. Revised and enlarged. By **Alvin A. Hubbell,** M.D., Ph.D., Professor of Clinical Ophthalmology in the University of Buffalo, etc. Duodecimo, pp. 197. Illustrated by selected portraits and cuts. Chicago: W. T. Keener & Company. (Cloth, $1.75 net.)

Dr. Hubbell, who for many years has been numbered among the prominent ophthalmologists of Buffalo, has performed a distinct service to medicine and medical men in presenting this historic sketch. Medical history, always interesting, is doubly so when one knows the historian, as in the present instance. It is of special importance that younger members of the profession should become familiar with American medical history. The topic of ophthalmology has been prepared by this author with painstaking care, reflecting credit alike to subject and historian. It is to be noted, in particular, that the book is embellished by portraits and cuts of institutions, thus giving an increased value and a more substantial character. Let us hope that the history of other medical specialties will be prepared by as competent historians and made as interesting.

